# Nanodiamond based surface modified screen-printed electrodes for the simultaneous voltammetric determination of dopamine and uric acid

**DOI:** 10.1007/s00604-019-3315-y

**Published:** 2019-02-22

**Authors:** Marina Baccarin, Samuel J. Rowley-Neale, Éder T. G. Cavalheiro, Graham C. Smith, Craig E. Banks

**Affiliations:** 1 Faculty of Science and Engineering, Manchester Metropolitan University, Chester Street, Manchester, M1 5GD UK; 2 Instituto de Química de São Carlos, Universidade de São Paulo, São Carlos, SP 13566-590 Brazil; 3 Manchester Fuel Cell Innovation Centre, Manchester Metropolitan University, Chester Street, Manchester, M1 5GD UK; 4 Faculty of Science and Engineering, Department of Natural Sciences, University of Chester, Thornton Science Park, Pool Lane, Ince, Chester, CH2 4NU UK

**Keywords:** Detection, Dopamine, Uric acid, Nanodiamonds, Screen-printed electrodes, Micro-electrode array, Electrochemistry, Electrocatalysis

## Abstract

The electroanalytical detection of the neurotransmitter dopamine (DA) in the presence of uric acid (UA) is explored for the first time using commercially procured nanodiamonds (NDs). These are electrically wired via surface modification upon screen-printed graphite macroelectrodes (SPEs). The surface coverage of the NDs on the SPEs was explored in order to optimize electroanalytical outputs to result in well-resolved signals and in low limits of detection. The (electro)analytical outputs are observed to be more sensitive than those achieved at bare (unmodified) SPEs. Such responses, previously reported in the academic literature have been reported to be electrocatalytic and have been previously attributed to the presence of surface sp^2^ carbon and oxygenated species on the surface of the NDs. However, XPS analysis reveals the commercial NDs to be solely composed of nonconductive sp^3^ carbon. The low/negligible electroconductivity of the NDs was further confirmed when ND paste electrodes were fabricated and found to exhibit no electrochemical activity. The electroanalytical enhancement, when using NDs electronically wired upon SPEs, is attributed not to the NDs themselves being electrocatalytic, as reported previously, but rather changes in mass transport where the inert NDs block the underlying electroactive SPEs and create a random array of graphite microelectrodes. The electrode was applied to simultaneous sensing of DA and UA at pH 5.5. Figures of merit include (a) low working potentials of around 0.27 and 0.35 V (vs. Ag/AgCl); and (b) detection limits of 5.7 × 10^−7^ and 8.9 × 10^−7^ M for DA and UA, respectively.

**Electronic supplementary material:**

The online version of this article (10.1007/s00604-019-3315-y) contains supplementary material, which is available to authorized users.

**Graphical abstract:**
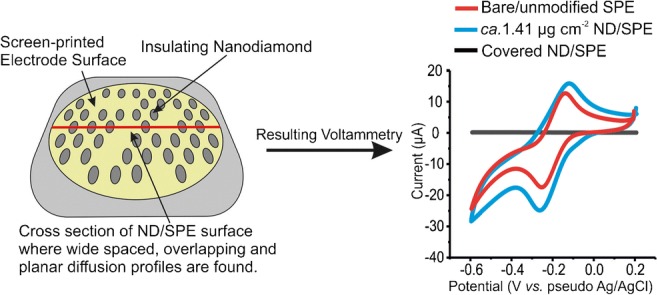
The electroanalytical enhancement of screen-printed electrodes modified with inert/non-conductive nanodiamonds is due to a change in mass transfer where the inert nanodiamonds facilitate the production of a random microelectrode array.

## Introduction

Dopamine (DA) is responsible for a number of functions in the human body correlated with movement, memory, emotional control, sleep and attention [16, 24]. It has been shown that abnormal levels of DA is a key indicator associated with certain neurological diseases, such as Parkinson’s, depression, and Alzheimer’s. As a result of this, research has focused upon finding ever increasingly accurate and rapid DA detection methods [20]. There are numerous studies within the literature that report the electroanalytical detection for the quantification of DA within biological and in-vivo samples [1, 9, 10, 13]. Electrochemical detection techniques are favoured by researchers due to their low cost, portability and analytical sensitivity towards a range of target analytes. There is however, an inherent problem faced using electroanalytical techniques towards the sensing of DA, as real samples usually coexist with uric acid (UA), which exhibits an electrochemical signal that overlaps with DA. Consequently, DAs analytical determination can become convoluted; researchers are constantly exploring different techniques and materials that are able to separate the analytical signals. Various approaches have been utilised in order to overcome this issue. For example Song et al. [27] reported that Fe_3_O_4_ nanoparticle/graphene oxide modified glassy carbon electrodes (GCE) were able to differentiate the peak signal outputs for DA and UA within urine, blood and brain tissue samples using differential pulse voltammetry (DPV), displaying low limits of detection (LODs) in pH 7 phosphate buffer for DA and UA of 5.3 × 10^−8^ and 4.1 × 10^−7^ M, respectively. Han and co-workers [6] utilised a modified GCE with a dispersion of chitosan-graphene for the simultaneous detection of ascorbic acid (AA), DA, and UA, which under optimal conditions, via DPV; the LODs were reported to correspond to 5.0 × 10^−5^ M, 1.0 × 10^−6^ M, and 2.0 × 10^−6^ M for AA, DA and UA, respectively (in pH 7 phosphate buffer).

Another approach has been to utilise nanodiamond (NDs). NDs were initially synthesized in the 1960s [11], and have since been used for a plethora of applications, such as the manufacture of micro abrasives, the production of cosmetics [31], delivery of drugs [32], and the development of electrochemical sensors; the latter is overviewed within Table 1. For example, Simioni et al. [26] modified a GCE with an aqueous ND dispersion (*note the NDs utilized in* Simioni et al. [26] *study were commercially procured from Sigma-Aldrich and are the same type of NDs utilized within this study*) and reported the modified electrode to exhibit improved heterogeneous electron transfer kinetics (*k*°) and a lower LOD (2.2 × 10^−7^ M) towards the sensing of pyrazinamide than that achievable at an unmodified GCE; Simioni et al. [26] attributed this increase in analytical sensitivity due to the NDs increasing the electrode area. The studies described within Table 1 are thorough in their approach at studying the properties of ND but they lacking with regard to the transferability of their results to “infield” biomedical scenarios as they utilize traditional laboratory based electrodes, e.g. glassy carbon (GC), gold and boron-doped diamond (BDD) as underlying electrode supports. All of these require several time consuming cleaning and polishing stages in between separate samples tests. The utilization of screen-printed electrodes (SPEs) as the underlying supporting electrode decreases the time necessary to perform separate tests, since SPEs are comparatively cheap, tailorable and disposable. Therefore, SPEs allow for rapid and repeated on-site testing [14]. Previous studies utilizing SPEs as supporting platforms for electrochemical sensors have been highlighted in ESI Table 1. Many of the studies that present cases of ND catalysis are intriguing but yet counterintuitive [22, 28], as bulk diamond is an inherent insulating material with a relatively large band gap of 5.47 eV [29]. It has however, been theorized that NDs exhibit useful electrochemical properties due to the presence of unsaturated bonding, sp^2^-like carbon and the formation of oxygenated species within/upon their outer structure [7] and a large surface to mass ratio. Holt et al. [7] used commercially sourced NDs (procured from Shenzhen Jingangyuan New Material Co., P. R. China.) which were shown to enhance the voltammetric outputs of ND modified gold electrodes towards the redox couples Ru(NH_3_)_6_^3+/2+^ and Fe(CN)_6_^4−/3–^. These reported electrocatalytic/electronic properties suggest that NDs are potentially exciting for the basis of electrochemical sensing platforms. Consequently, we re-evaluate the electroanalytical performance of ND/SPEs for the simultaneous detection of DA and UA where enhanced voltammetric signals are observed using ND modified SPEs over bare SPEs; in contrary to the academic literature we provide a different insight into the reported and observed (in our work) the beneficial electroanalytical outputs.

**Table 1:** Comparison of different electrodes modified with NDs used for the detection of a range of target analytes

Modifier material	Bare electrode	Analyte	LOD (M)	Reference
PAA/N-NCD/GOx	gold electrode	Glucose	5.0 × 10^−6^	Zhao et al. [34]
Chit/UND/Hb	GCE	H_2_O_2_	4.0 × 10^−7^	Zhu et al. [35]
ND-NS(HRP)	GCE	H_2_O_2_	5.9 × 10^−5^	Gopalan et al. [5]
QAS-ND/Mb	GCE	H_2_O_2_	3.5 × 10^−6^	Xiao-Ling et al. [33]
LOx/DNPs	gold electrode	Lactate	1.5 × 10^−5^	Briones et al. [2]
Ni-NDs	BDD	Glucose	5.0 × 10^−8^	Dai et al. [4]
ND-DHP	GCE	Codeine	5.5 × 10^−8^	Simoni et al. [25]
ND	GCE	Pyrazinamide	2.2 × 10^−7^	Simioni et al. [26]
ND	SPE	DA, UA	5.7 × 10^−7^, 8.9 × 10^−7^	This work

*Key*: *PAA/N-NCD/GOx* poly(allylamine hydrochloride/ non-doped nanocrystalline diamond/ glucose oxidase, *Chit/UND/Hb* chitosan/ undoped nanocrystalline diamond/ hemoglobin, *GCE* glassy carbon electrode, *ND-NS(HRP)* nanodiamond-based sponges with entrapped horseradish peroxidase, *QAS-ND/Mb* quaternary ammonium salt-nanodiamond/myoglobin, *LOx/DNPs* Lactate oxidase /undoped diamond nanoparticles, *Ni-ND* nicklel-nanodiamond, *BDD* boron-doped diamond, *ND-DHP* nanodiamond-dihexadecyl phosphate, *ND* nanodiamond, *DA* dopamine, *UA* uric acid

## Experimental

All chemicals (analytical grade or higher) were used as received from Sigma-Aldrich without any further purification, which includes the nanodiamonds (NDs) [23]. The NDs are electrochemically wired via surface modification (drop-casting) upon SPEs, (see below). All solutions were prepared with deionised water of resistivity not less than 18.2 MΩ cm and were vigorously degassed prior to electrochemical measurements with high purity, oxygen free nitrogen.

Electrochemical measurements were performed using an Ivium Compactstat™ (Netherlands) potentiostat. All measurements were conducted, using a conventional three-electrode system utilizing a pseudo Ag/AgCl as a reference and a carbon ink formulation as a counter and working electrodes. The SPEs have a 3.1 mm diameter working electrode and were fabricated in-house with the appropriate stencils using a DEK 248 screen-printing machine (DEK, Weymouth, U.K.) [3]. A full description of the SPE production methodology is found within the supporting information. The bare/unmodified SPEs fabricated display a heterogeneous electron transfer rate constant, *k*^0^, as measured using the Nicholson’s [17], and Lavagnini et al. [12] methods with [Ru(NH_3_)_6_]^3+/2+^ which is found to correspond to 4.2 × 10^−3^ cm∙s^−1^. The SPEs were modified with NDs via drop-casting. This procedure entials, 1 mg of NDs (optimized value) were solubilized in 1.0 mL deionized water and then, from this dispersion, 8 μL were deposited onto the SPEs surface. After 30 min, the solvent is completely evaporated (at ambient temperature) and the ND/SPEs are ready for use. Note that in addition to the SPEs GC (3 mm diameter, BAS, USA), edge plane pyrolytic graphite (EPPG Le Carbone, Ltd. Sussex, UK; 4.9 mm diameter) and carbon paste electrodes (the technique by which they were fabricated is reported within the SI) were utilized.

## Results and discussion

### Physicochemical characterisation of the nanodiamonds (NDs)

The physicochemical characterisation of the NDs was undertaken in order to independently determine their quality and purity. This included Raman spectroscopy, scanning electron microscope (SEM), transmission electron microscopy (TEM), X-ray photoelectron spectroscopy (XPS) and X-ray diffraction (XRD). A full description of the performed physicochemical characterisation and equipment utilised can be found within the ESI (see Figure S1, S2 and S3). From the performed physicochemical analysis, we can be confident that the NDs utilized throughout this study are of both a high purity and quality.

### Electroanalytical performance of the ND/SPEs

**Fig. 1:**
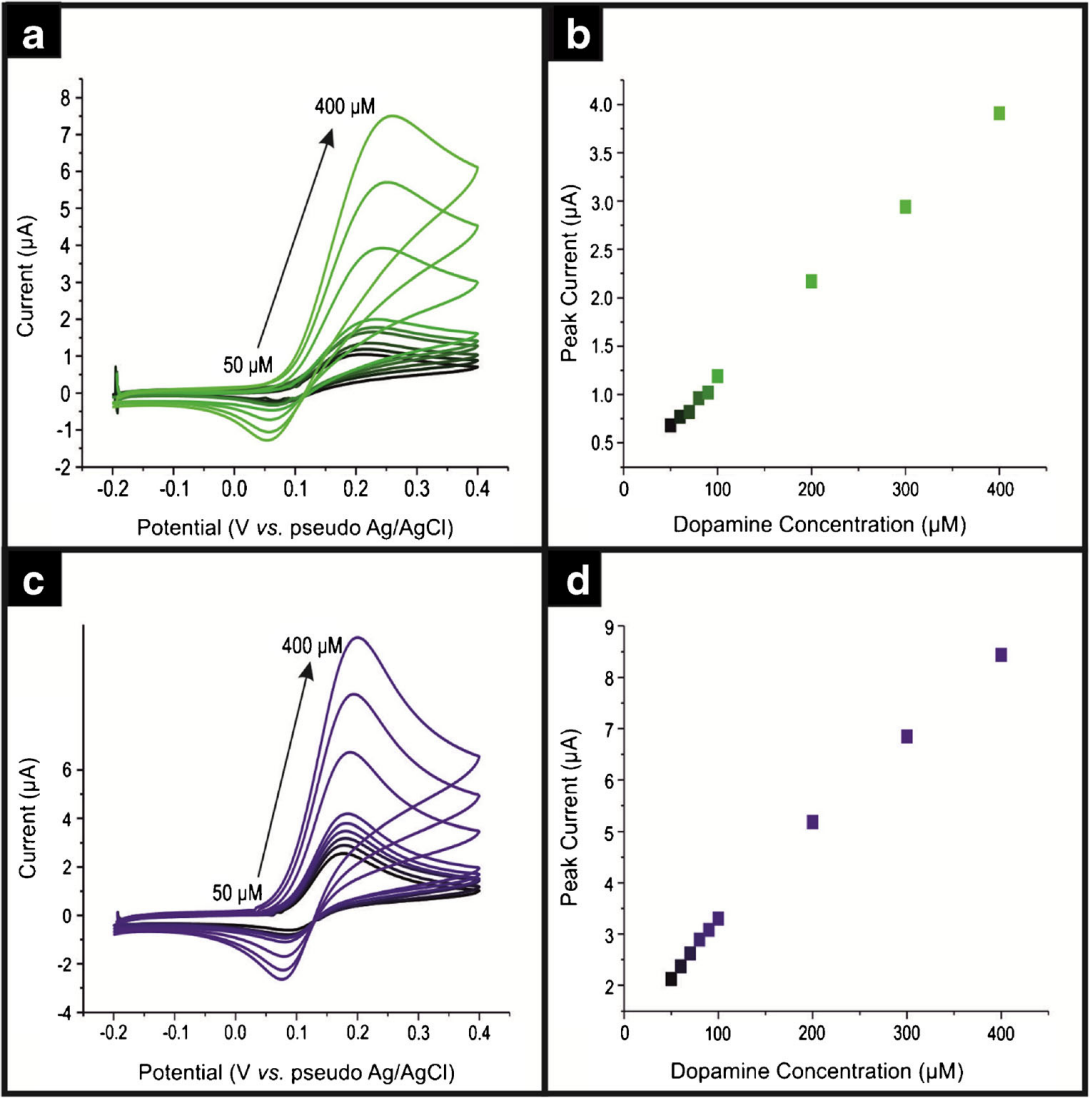
Typical cyclic voltammograms using bare screen printed electrodes (SPEs) (**a**) and a ca. 140 ng**∙**cm^−**2**^ ND/SPE (**c**) over a range of DA concentrations from 50 to 400 μM in pH 7.4 phosphate buffer . Scan rate: 50 mV**∙**s^−**1**^. The analysis of voltammetric peak current as a function of concentration is shown in the respective (**b**) and (**d**)

**Fig. 2:**
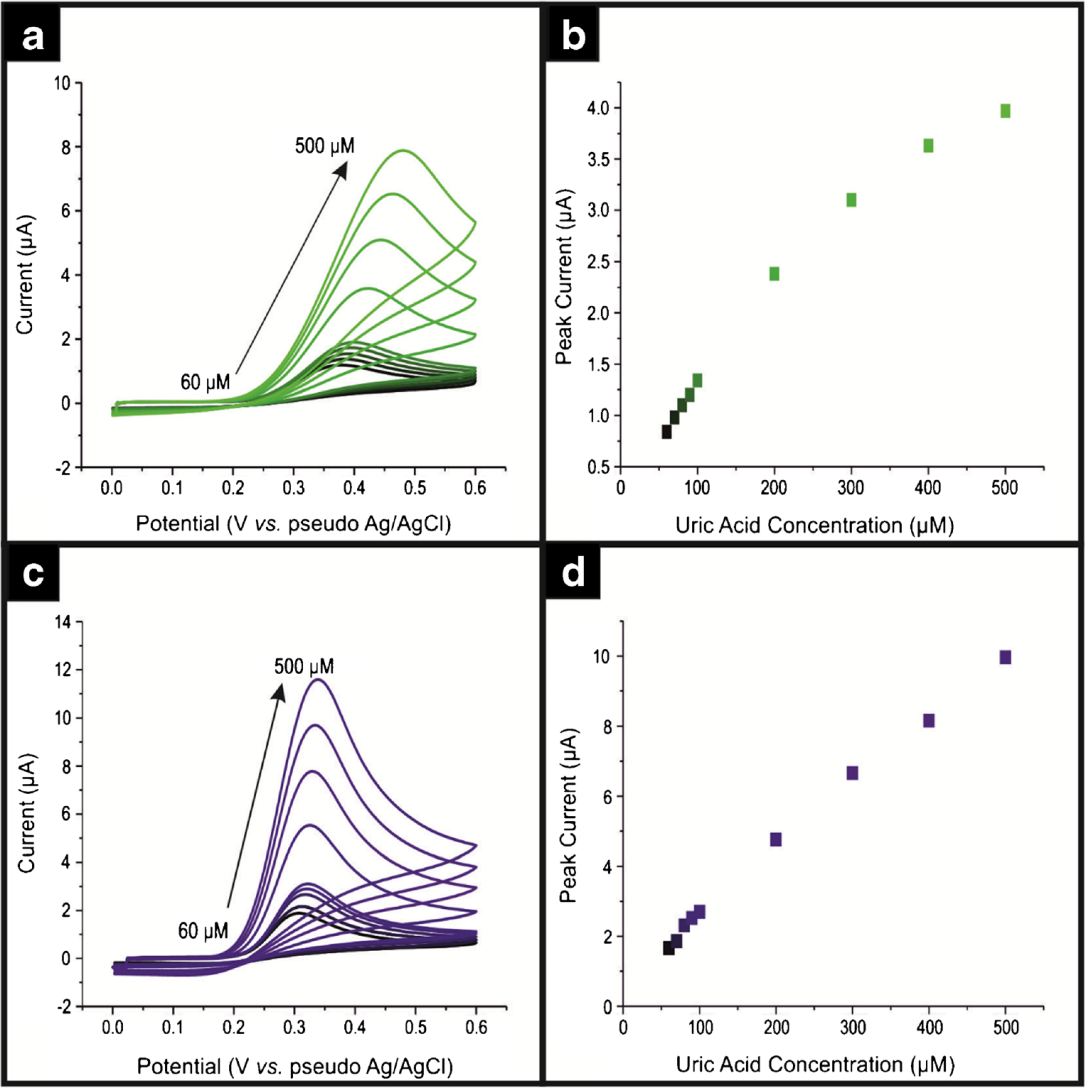
Typical cyclic voltammograms using SPE (**a**) and a ca. 140 ng∙cm^−2^ ND/SPE (**b**) over a range of UA concentrations from 60 to 500 μM in pH 7.4 phosphate buffer **.** Scan rate: 50 mV∙s^−1^. The analysis of voltammetic peak current as a function of concentration is shown in the respective (**b**) and (**d**)

Initially the electrochemical oxidation of DA (Fig. 1) and UA (Fig. 2) was explored using bare/unmodified SPEs and ND surface modified SPEs (ND/SPEs) over a range of DA and UA concentrations. Note the use of SPEs as substrates for sensing devices has numerous advantages over more traditional carbon based electrodes as they are cheap, reproducible and disposable, making them perfect for in-field use where a single shot sensor which has no memory effect, is desirable. Clear and distinct voltammetric profiles are evident at both the bare SPEs and the ND/SPEs. Figure 1c and 2c display that for DA and UA the analytical signals appear at +0.18 V and + 0.31 V, respectively, giving an approximate peak separation of 130 mV, clearly demonstrating that the simultaneously detection of DA and UA is possible using the ND/SPEs. A closer inspection of the voltammetric peak currents/signals of DA and UA are observed to be relatively increased using the ND/SPEs over that of the bare SPEs (see Figs. 1 and 2); this observation resonates with the academic literature (see Table 1).

The detection parameters for DA and UA can be optimized by evaluating the p*K*_a_ values and adjusting the pH of the electrolyte solution in accordance with the Henderson-Hasselbach equation [15]. Given the p*K*_a_ of UA is 5.50 it can be expected to be a charge neutral species within the pH ca. 7 solutions, however at pH values of ca. 5, UA is expected to exist in its acidic form. DA which has p*K*_a_ value of 8.9, will only exist in its acidic form when UA does not, as the combination of both molecules will affect the acid/base equilibrium. The electroanalytical response using the ND/SPEs is shown within Figure S4. It is clear that the greatest separation between the DA and UA oxidation signals/peaks was acquired utilizing a pH 5.5 solution. Figures S5 demonstrates that a pH 5.5 acetate buffer gives rise to larger analytical signals/peaks over that of a pH 5.5 phosphate buffer solution (see Figure S6). Using the optimized conditions, the performance of the ND/SPEs towards the analytical sensing of DA and UA was evaluated. Note that a range of different surface modifications of NDs upon the supporting SPEs were explored and monitored as a function of analytical output verses ND coverage (ng∙cm^−2^), where increasing mass coverages of NDs result in larger analytical peaks/signals until a critical mass coverage is achieved after which, further coverages reduce the achievable analytical signal. It was found that an optimal response is achievable at a coverage of NDs of ca. 140 ng∙cm^−2^ (see Table S2).

**Fig. 3:**
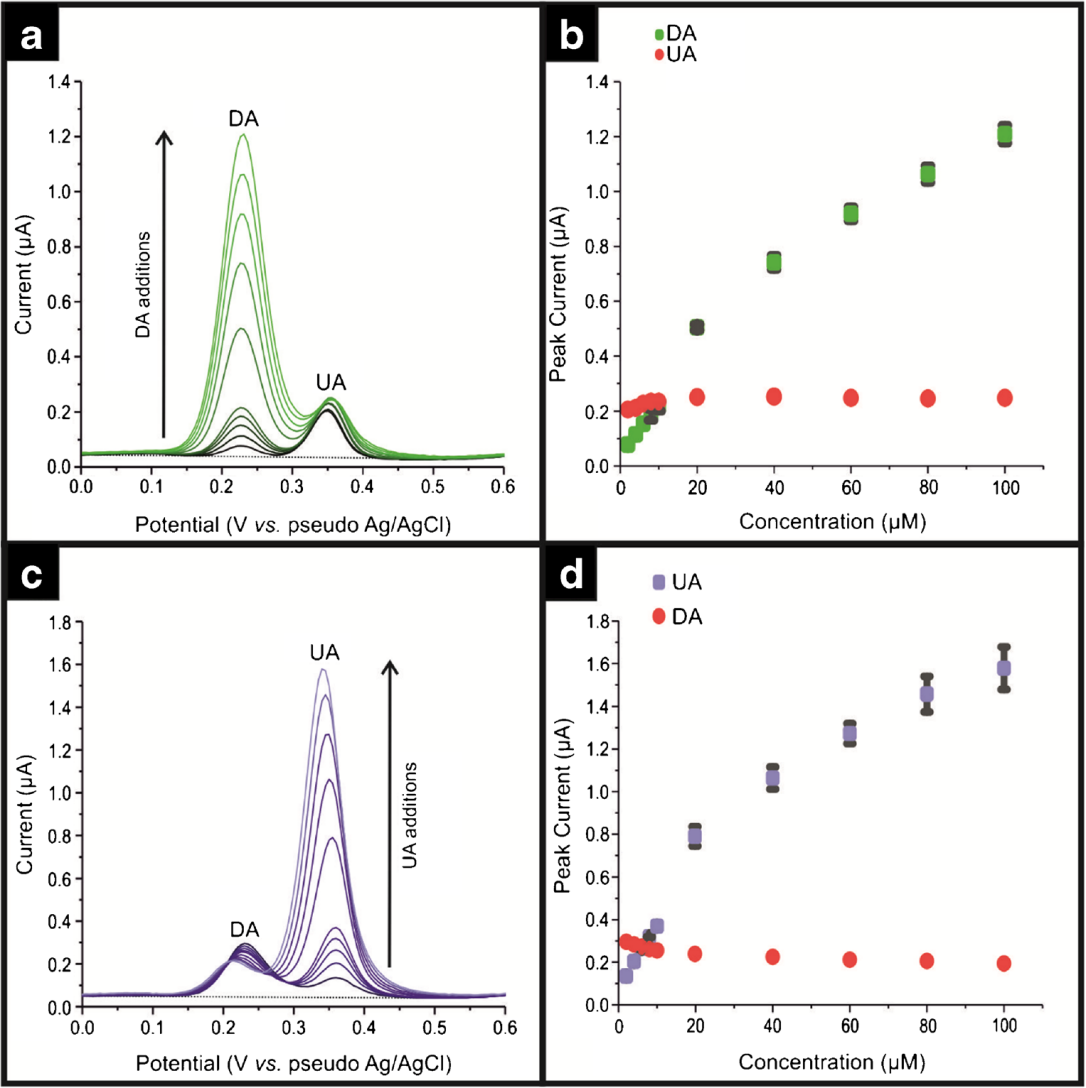
Differential pulse voltammograms (DPV) using ca. 140 ng∙cm^−2^ for: **a** DA concentrations from 2 to 100 μM with 20 μM UA fixed; **c** UA concentrations from 2 to 97 μM with 20 μM DA fixed and the respective analytical curve for different additions of DA in (**b**) and for UA in (**d**). Each point is the average of three measurements and standard deviation. All measurements were performed in pH = 5.5 acetate buffer . Parameters of DPV: E-pulse = 20 mV; t-pulse = 200 ms; equivalent scan rate: 10 mV∙s^−1^

The electroanalytical performance of the ND/SPE is shown in Fig. 3 where increasing concentrations of DA in the presence of a fixed concentration of UA are explored and then the reverse is done, where the concentration of UA is fixed in a fixed concentration of DA. Clear separation of the DA and UA analytical signals/peaks. The analysis of the voltammetric peak currents as a function of DA and UA concentrations is shown in Fig. 3b and d with the following lines of best fit for the detection of DA and UA. In the case of DA the line equation was *I*/*A* = 4.1 × 10^−8^ A + 0.02 AM^−1;^ *N = 5*, *R*^*2*^ = 0.99 and *I*/*A* = 2.2 × 10^−7^ A + 0.01 AM^−1^; *N = 5; R*^*2*^ = 0.96. The initial linear range was used to calculate the LOD (*3σ*) of DA, which is found to be 0.57 μM. In the case of UA the line equations corresponded to *I*/*A* = 8.2 × 10^−8^ A + 0.030 AM^−1^; *N = 5*; *R*^*2*^ = 0.99 and *I*/*A* = 4. 9 × 10^−7^ A + 0.011 AM^−1^; *N = 5; R*^*2*^ = 0.96 m, respectively. The initial linear range was utilized in order to obtain the LOD (*3σ*) for UA of 0.89 μM. Note that the percentage standard deviation bars for the DA and UA signal can be observed in Fig. 3b and d, respectively. In both cases there was an increase in *σ* as concentration increased, the low percentage variation attests to the reproducibility of SPEs.

### Understanding the enhanced electroanalytical performances using ND/SPEs

**Fig. 4:**
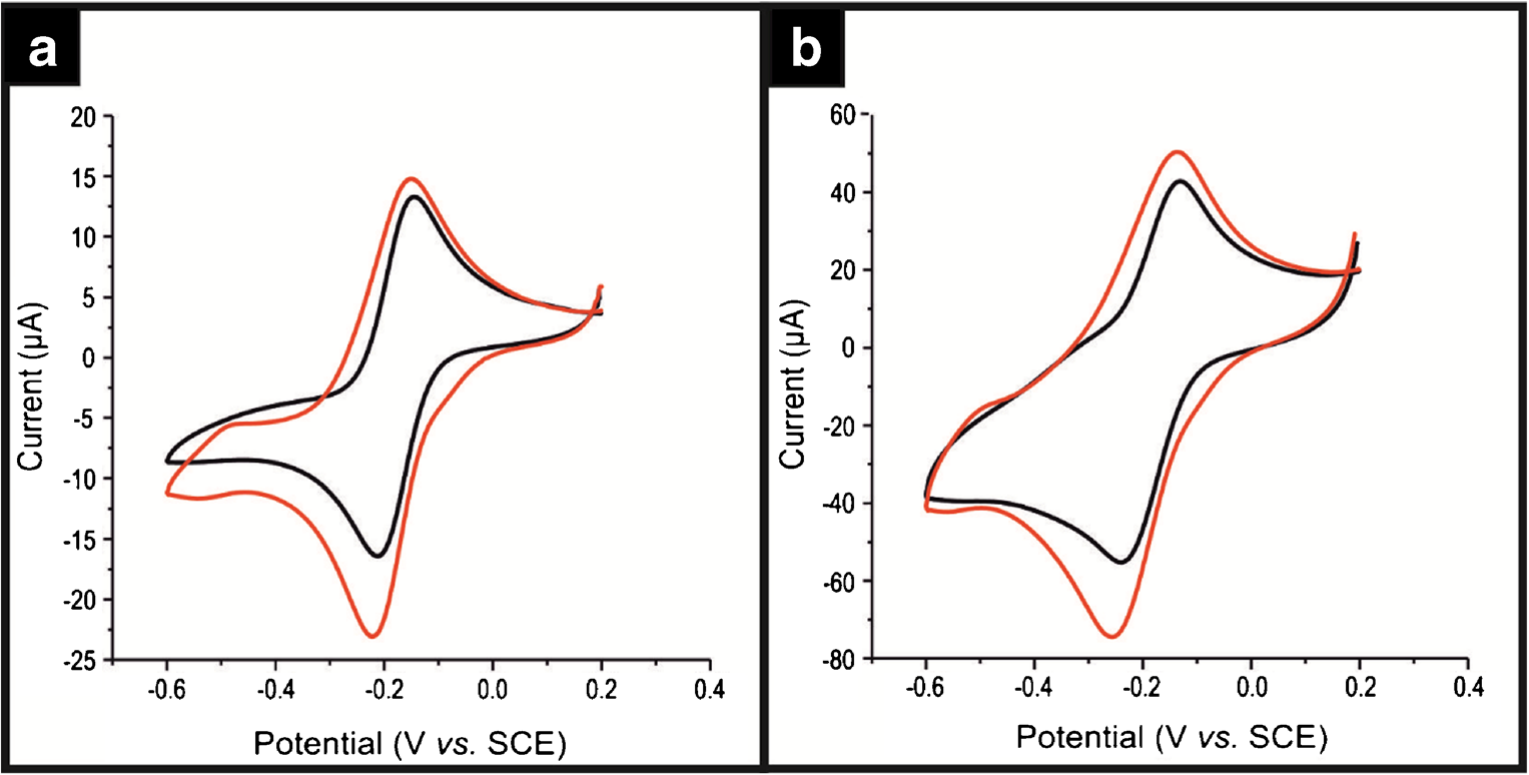
Typical cyclic voltammograms recorded in 1 mM [Ru(NH_3_)_6_]^3+/2+^ / 0.1 M KCl for a bare unmodified (black line) GCE (**a**) and EPPG (**b**) modified with ca. 140 ng∙cm^−2^ NDs (red line). Scan rate: 100 mV∙s^−1^

**Fig. 5:**
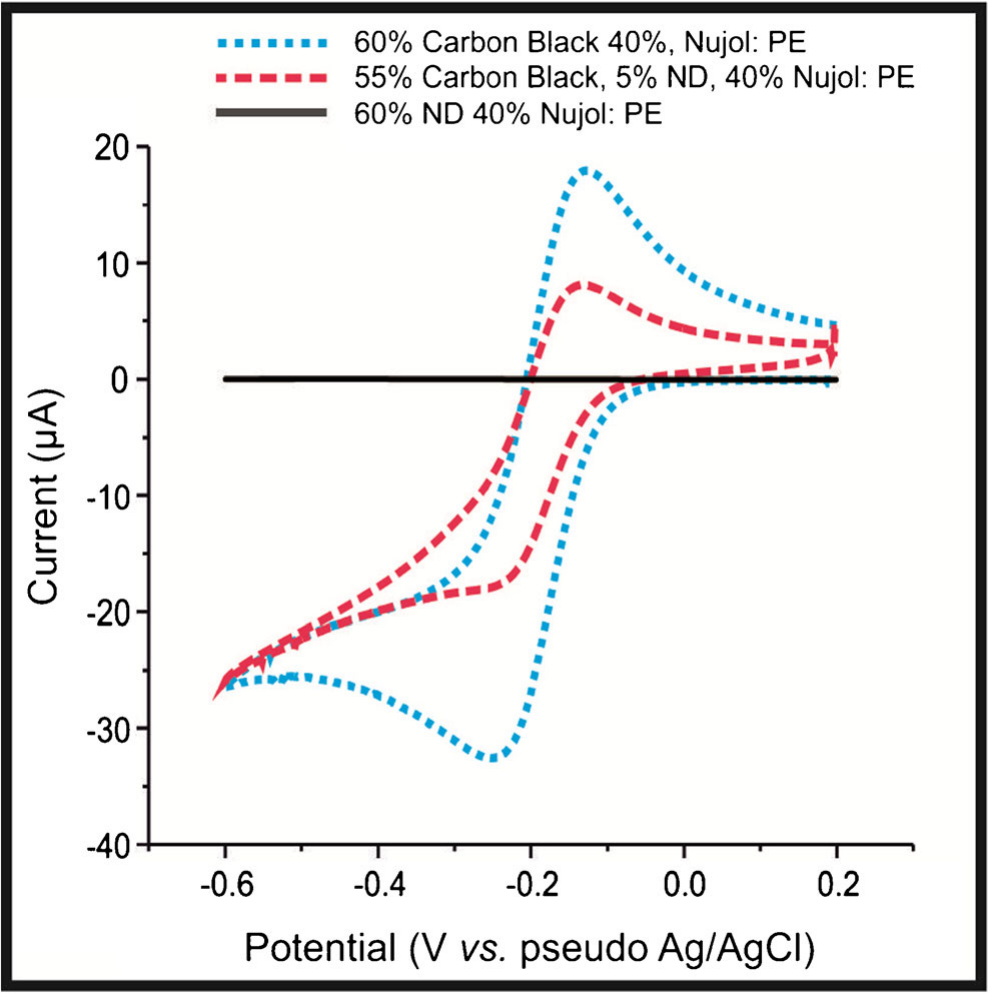
Cyclic voltammograms for carbon paste electrodes (PE) with increasing amount of ND in 1 mM [Ru(NH_3_)_6_]^3+/2+^ / 0.1 M KCl; Scan rate: 50 mV∙s^−1^. Electrode compositions: (carbon black (60%): nujol (40%)) (small dotted line), 60: 40% (NDs: nujol) (solid line), and (carbon black (55%): NDs (5%): nujol (40%)) (large dotted line) for 1 mM [Ru(NH_3_)_6_]^3+/2+^ / 0.1 M KCl

**Fig. 6:**
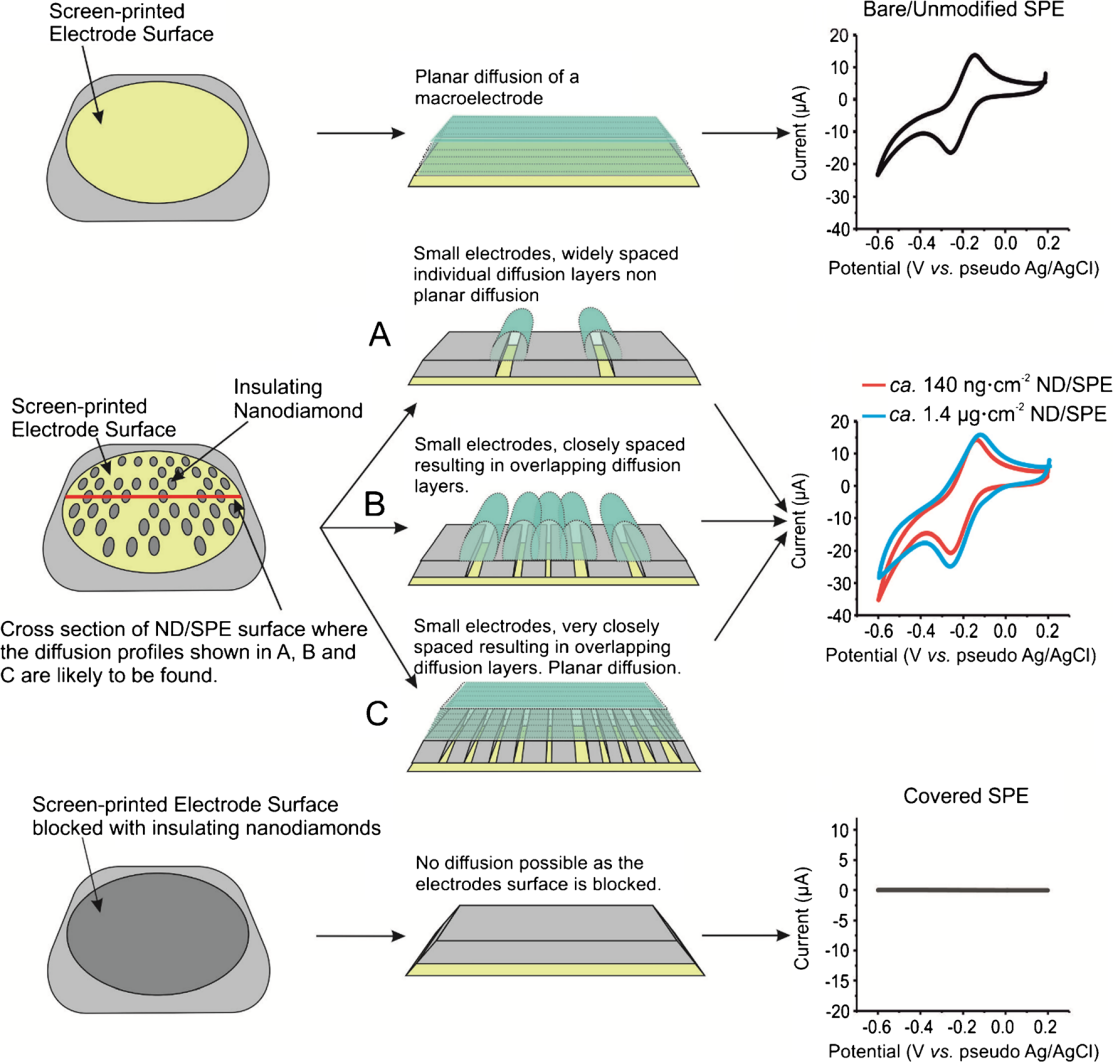
Cyclic voltammograms for a bare/unmodified SPE and then following surface modification with ca. 140 ng∙cm^−2^ and ca. 1.4 μg∙cm^−2^ of NDs, in 1 mM [Ru(NH_3_)_6_]^3+/2+^ / 0.1 M KCl. Scan rate: 50 mV∙s^−1^. One can clearly see how the voltammetric response changes as the coverage of NDs is increased up to a point where the electrode surface is full covered/blocked and electron transfer is not possible resulting in no voltammetric signals. Note in reality the blocking NDs are randomly distributed upon the electrode surface

The ability of a ND modified SPE to simultaneously detect UA and DA suggests that the NDs are “electrocatalytic” towards DA and UA. Indeed the elegant work of Holt and co-workers [7, 8, 18, 21]. suggests that electron transfer can occur at the surface of undoped NDs due to the presence of a complex arrangement of sp^2^ and sp^3^ carbon, which facilitates electron transfer between the NDs and the electrolyte [18]. In order to explore this further, the ND/SPEs were benchmarked using the near ideal outer-sphere probe, [Ru(NH_3_)_6_]^3+/2+^ and for comparative purposes, GCE and EPPG were also modified with NDs of varying coverages. Figure 4 shows the responses of the ND modified GCE and EPPGs, which clearly demonstrates some intriguing voltammetry, where in addition to the expected redox probe responses; two additional waves are seen prior to the main voltammetric redox response. The initial reduction peak is observed at −217 mV and – 255 mV (vs. pseudo Ag/AgCl) for the ND/GCE and ND/EPPG, respectively. In both cases secondary voltammetric peaks are observed at ca. – 570 mV (vs. pseudo Ag/AgCl). Note that our experimental observations agree with the theory presented by Ward et al. [30] who demonstrate via numerical simulations that modifying an electrode surface with inert/less electrochemically active materials the electrode surface becomes electrochemically heterogeneous with two distinct zones that differ in their rate constants from *fast* to *slow*. This results in split peak voltammetry where two peaks would be observed rather than the expected redox process. Interestingly, SPEs do not display this secondary peak when modified with the analytically optimal coverage of ca. 140 ng cm^−2^ NDs (see Table S2). This is likely due to the differing surface roughness [19], where SPEs have a relatively rougher surface than that of GCE or EPPG (see Figure S7 and SI), or the scan rate utilized was not sufficiently fast to display split peak volammetry in the case of SPEs. To further consider the “electrocatalytic” activity of the NDs (see above), carbon paste electrodes were fabricated using varying amounts of NDs. Figure 5 shows the CVs pertaining to three carbon paste variants, those being; 60% carbon black and 40 nujol, 60% ND and 40% nujol, and 55% carbon black, 5% ND and 40% nujol. The differing voltammetric responses demonstrate that upon incorporation of an increasing amount of ND into the carbon paste electrode results in a decrease in electrochemical activity/ electron transfer and ultimately results in complete loss of electron transfer; such observations suggest that the NDs are non-conductive. As shown in Fig. 6 we also explore the effect of varying the coverages of NDs, via surface modification upon SPEs, where initially an improvement in electrochemical activity is observed, after which, the response decreases to a point where no electrochemical activity is observed. In all cases, a plot of peak height against the square root of scan rate yielded a linear relationship, thus implying the mass transport occurring at the bare/unmodified SPEs and ND modified SPEs is due to diffusional processes and not a thin layer effect. While the reports of Holt et al and others maybe plausible, we suggest, this is not credible, given the observed voltammetric profiles (i.e. towards the DA, UA, Ru(NH_3_)_6_]^3+/2+^ and incorporated within a carbon paste electrode) and that physicochemical characterisation, verifies that the NDs are solely composed of insulating sp^3^ carbon (see SI and Figure S8)). Given the above, rather than simply attribute the beneficial electroanalytical responses of the ND/SPEs to false “electrocatalysis”, the voltammetric responses coupled with the thorough physicochemical analysis indicate that instead, there is a change in the mass transport. Where the incorporation of insulating/non-conductive NDs, either in the bulk of, or on the surface, of an electrode, produce a randomly distributed graphite microelectrode array, since the inert/non-conductive NDs block the underlying electroactive electrode surface.

## Conclusions

We have explored the use of ND modified SPE as the basis of an electrochemical sensing platform towards the sensing of DA and UA. We observe an electroanalytical enhancement using NDs over that of bare/unmodified SPEs, indicating a potential “electrocatalysis”. However, further electrochemical and physicochemical characterization verifies that NDs are inert/non-conductive. Thus in summary, the observed beneficial electroanalytical response within the academic literature (see Table 1) is not due to the NDs being “electrocatalytic” but rather a change in mass transfer where the inert NDs facilitate the production of a random microelectrode array; the implications for previous observations (i.e. Table 1) are justified.

## Electronic supplementary material

ESM 1 (DOCX 5253 kb)
