# Supplementary material for: Nanodiamond based surface modified screen-printed electrodes for the simultaneous voltammetric determination of dopamine and uric acid
Source: Mikrochim Acta. 2019 Feb 22;186(3):200. doi: 10.1007/s00604-019-3315-y (PMC6394810; doi:10.1007/s00604-019-3315-y)
Supplement: Supplementary file 1 — (DOCX 5253 kb) [file 604_2019_3315_MOESM1_ESM.docx]

**Electronic Supporting Information:**

**Nanodiamond based surface modified screen-printed electrodes for the simultaneous voltammetric determination of dopamine and uric acid**

Marina Baccarin,^1,2^ Samuel J. Rowley-Neale,^1,3^ Éder T. G. Cavalheiro,^2^ Graham C. Smith^4^ and Craig E. Banks^1,3*^

*^1^: Faculty of Science and Engineering, Manchester Metropolitan University, Chester Street, Manchester M1 5GD, UK.*

^2^: *Instituto de Química de São Carlos, Universidade de São Paulo, 13566-590, São Carlos, SP, Brazil*

*^3^: Manchester Fuel Cell Innovation Centre, Manchester Metropolitan University, Chester Street, Manchester M1 5GD, UK.*

*^4:^ Faculty of Science and Engineering, Department of Natural Sciences, University of Chester, Thornton Science Park, Pool Lane, Ince, Chester CH2 4NU, UK*

*To whom correspondence should be addressed.

Email: [c.banks@mmu.ac.uk](mailto:c.banks@mmu.ac.uk); Tel: ++(0)1612471196; Fax: ++(0)1612476831

Website: [www.craigbanksresearch.com](http://www.craigbanksresearch.com)

**Screen-printed Electrode production**

The working electrodes were screen-printed graphite electrodes (SPE), which have a 3.1 mm diameter working electrode. The SPEs were fabricated in-house with the appropriate stencils using a DEK 248 screen-printing machine (DEK, Weymouth, U.K.).^1^ These electrodes have been used extensively in previous studies.^2-6^ In their fabrication; first a carbon-graphite ink formulation (product code C2000802P2; Gwent Electronic Materials Ltd., U.K.) was screen-printed onto a polyester (Autostat, 250 μm thickness) flexible film This layer was cured in a fan oven at 60 °C for 30 minutes. Next, a silver/silver chloride reference electrode was included by screen-printing Ag/AgCl paste (product code C2030812P3; Gwent Electronic Materials Ltd., U.K.) onto the polyester substrates and a second curing step was undertaken where the electrodes were cured at 60 °C for 30 minutes. Finally, a dielectric paste (product code D2070423D5; Gwent Electronic Materials Ltd., U.K.) was then printed onto the polyester substrate to cover the connections. After a final curing at 60 °C for 30 minutes the SPEs are ready to be used and were connected *via* an edge connector to ensure a secure electrical connection.^7^ The unmodified SPEs have been reported previously and shown to exhibit a heterogeneous electron transfer rate constant, *k^o^,* of *ca*. 10^–3^ cm s^–1^, as measured using the [Ru(NH_3_)_6_]^3+/2+^ outer-sphere redox probe.^8^ The ND ink was fabricated by incorporating ND into an graphite ink on the basis of the weight percent of *M*_P_ to *M*_I_, where *M*_P_ is the mass of ND particulates and *M*_I_ is the mass of the ink formulation used in the printing process, *i.e*. % = (*M*_P_ / *M*_I_ ) × 100.

**Physicochemical Equipment Specification**

Physicochemical characterisation of the NDs was performed. Scanning electron microscope (SEM) images were obtained using a JEOL JSM-5600LV model SEM. Transmission electron microscopy (TEM) images were obtained using a 200 kV primary beam under conventional bright-field conditions. The sample was dispersed onto a holey-carbon film supported on a 300 mesh Cu TEM grid. The X-ray photoelectron spectroscopy (XPS) data was acquired using a bespoke ultra-high vacuum system fitted with a Specs GmbH Focus 500 monochromated Al Kα X-ray source, Specs GmbH Phoibos 150 mm mean radius hemispherical analyser with 9-channeltron detection, and a Specs GmbH FG20 charge neutralising electron gun.^9^ X-ray diffraction (XRD) was performed using an “X'pert powder PANalytical” model with a copper source of Kα radiation (of 1.54 Å) and Kβ radiation (of 1.39 Å), using a thin sheet of nickel with an absorption edge of 1.49 Å to absorb Kβ radiation. A reflection transmission spinner stage (15 rpm) was implemented to hold the sample.

**Physicochemical Characterization of the NDs**

Figure 1 and 2 depict typical SEM and TEM images of the NDs indicating that they have an average diameter of 5 nm, which is in agreement with that stated by the supplier of 5 nm on average. Clearly, there are also distinct signs of agglomeration where the NDs form larger ND clusters (*i.e.* Figure 1A). Raman analysis of the NDs can be observed in ESI Figure S1 where the characteristic D and G bands can be observed at 1328 and 1637 cm^−1^, respectively. The D peak is representative of the bulk crystal whilst the G band is the split-interstitial defect of the diamond lattice.^10^ XRD analysis of the NDs is shown within Figure 1(B) where the characteristic peaks associated with the normal structure of ND are observed at *ca*. 44º, 75º and 92º, which correspond to the (111), (220) and (311) reflections.^11^ The XPS spectra for the NDs is shown in Figure 1(C) and (D). The concentration of approximately 93.5 atom percentage appears to contradict the independent reported specification of > 97% purity, as quoted by the supplier, however the stated purity is likely on a weight % basis and additionally XPS data is biased towards the surface where atmospheric contaminants such as oxides etc. are concentrated. For example, in the case of diamond, approximately 63% of the XPS signal intensity will be from within a depth of approximately 3.5 nm within the surface. Low levels of surface species were found, including carbon-bonded oxidized sulphur groups and nitrogen in the form of carbon-nitrogen bonds and (at a low level) quaternary ammonium-like ions. These are likely due to atmospheric contaminants and are observed due to the surface bias of the XPS technique. Further XPS analysis reveal critical insights in the carbon structure of the NDs with the results being presented later within the manuscript. From the above physicochemical analysis, we can be confident that the NDs utilized throughout this study are of both a high purity and quality.

**Carbon Paste Electrode Preparation**

Carbon paste electrodes were fabricated by mixing carbon black, nujol and ND in a pestle and mortar at the following specific ratios: 60% carbon black and 40 nujol, 60% ND and 40% nujol, and 55% carbon black, 5% ND and 40% nujol. The resultant paste was then inserted into a PTFE sleeve, which has a 3 mm diameter. An electrical contact was formed using a copper wire. The exposed surface of the paste electrodes was smoothed and rinsed with deionized water, after which they were ready for use.

**The Electrode Roughness**

The SPEs are shown to have a relatively higher SA (arithmetic average values of absolute height over whole surface) values of 1539.6 nm compared to 19.9 nm and 12.3 nm for EPPG and GCE, receptively. We infer this difference in surface roughness would result in grooves of the SPE such that a larger mass of NDs is reported to block the electroactive active graphitic areas. Theoretical work to investigate this hypothesis would be of interest. This was determined using white light profilometry (a ZeGage 3D Optical Surface Profiler, produced by Zygo) and based upon an average of 3 randomly selected areas upon the respective electrodes surface (see Figure S9).

**XPS analysis of the commercially procured NDs**

XPS analysis of the NDs supports this analysis as seen in Figure S10(A), which show the C KVV X-ray excited Auger peaks. The width of the main feature varies in a systematic manner according to the relative proportions of sp^2^ (graphitic) and sp^3^ (tetrahedral) bonding. A calibration of which has been published by J.C. Lascovich and S Scaglione.^12^ These authors differentiate the peak and determine the energy difference between the positive-going and the negative-going excursions. A low value of approximately 14 eV corresponds to 100% sp^3^ and a high value of approximately 23 eV corresponds to 100% sp^2^. The results of differentiation of our data are shown in Figure S10(B). Although there is some noise present, the separation is clearly in the region of 14 eV and indicates a 100% sp^3^ tetrahedrally-bonded material. Note that the kinetic energy of these Auger peak structures is in the region of 260 eV, therefore very surface sensitive. The carbon 1s peak was relatively broad and featureless and is shown fitted with a single Gaussian peak. The match is not perfect near the top of the peak, however, this may be an artefact due to slightly imperfect charge neutralisation during the measurement. There is no evidence for the presence of separate sp^2^ and sp^3^ components, and also very little evidence for high levels of oxygen-bonded components. It can therefore be asserted that the structure of the ND was 100% sp^3^, *i.e.* tetrahedrally bonded as one would expect for classical diamond structure; there was no evidence for a sp^2^ bonded surface layer.

**Table S1.** Comparison of studies that utilise SPEs and non-traditional electrode

supports towards the detection of DA and UA.

| Sensor | Linear range (µM) | | LOD (µM) | | Reference |
| --- | --- | --- | --- | --- | --- |
|  | DA | UA | DA | UA |  |
| In3C-GC | 10 – 100 | 10 – 100 | 1.70 | 4.99 | ^13^ |
| NiO/GR/SPE | 1 – 500 | 125 – 250 | 0.31 | – | ^14^ |
| Pd-NP/CPE | 0.1 − 151 | 0.5 − 225 | 0.03 | 0.15 | ^15^ |
| PG/GCE | 5 − 710 | 6 − 1330 | 2.0 | 4.8 | ^16^ |
| CILE | 2 − 1500 | 2 − 220 | 1.0 | 1.0 | ^17^ |

**Key**: In3C-GC; Indole-3-carboxaldehyde - glassy carbon electrode, Au/RGO/GCE; Au nanoplates/reduced graphene oxide/glassy carbon electrode, NiO/GR/SPE; graphene nanosheets/NiO nanoparticles/carbon screen printed electrode, Pd-NP/CPE; palladium nanoparticles/carbon paste electrode, PG/GCE; pristine graphene/glassy carbon electrode, CILE; carbon ionic liquid electrode.

**Table S2.** The ΔEP values at 100 mV s^-1^ and the *k*^o^ (deduced from the 1mM [Ru(NH_3_)_6_]^3+/2+^ scan rate studies) for a bare/unmodified SPE and the ND/SPE variants using the redox probes 1 mM [Ru(NH_3_)_6_]^3+/2+^ / 0.1 M KCl.

| SPE Variant | *kº* (cm s^−1^) |
| --- | --- |
|  |  |
| Bare/Unmodified SPE | 4.20 × 10^−3^ |
| 14 ng cm^−2^ ND/SPE | 4.43 × 10^−3^ |
| 140 ng cm^−2^ ND/SPE | 4.96 × 10^−3^ |
| 1.4 µg cm^−2^ ND/SPE | 4.84 × 10^−3^ |
| 7.1 µg cm^−2^ ND/SPE | 4.65 × 10^−3^ |
| 11.3 µg cm^−2^ ND/SPE | 3.97 × 10^−3^ |
| 14.1 µg cm^−2^ ND/SPE | 3.89 × 10^−3^ |
| 21.2 µg cm^−2^ ND/SPE | 3.92 × 10^−3^ |
| 28.2 µg cm^−2^ ND/SPE | 3.78 × 10^−3^ |

Figure S1. Physicochemical characterization of the commercially sourced NDs: (A) SEM (scale bar: 100 nm); (B) XRD; (C); XPS and (D) a high resolution selected portion of the XPS showing the carbon peak.


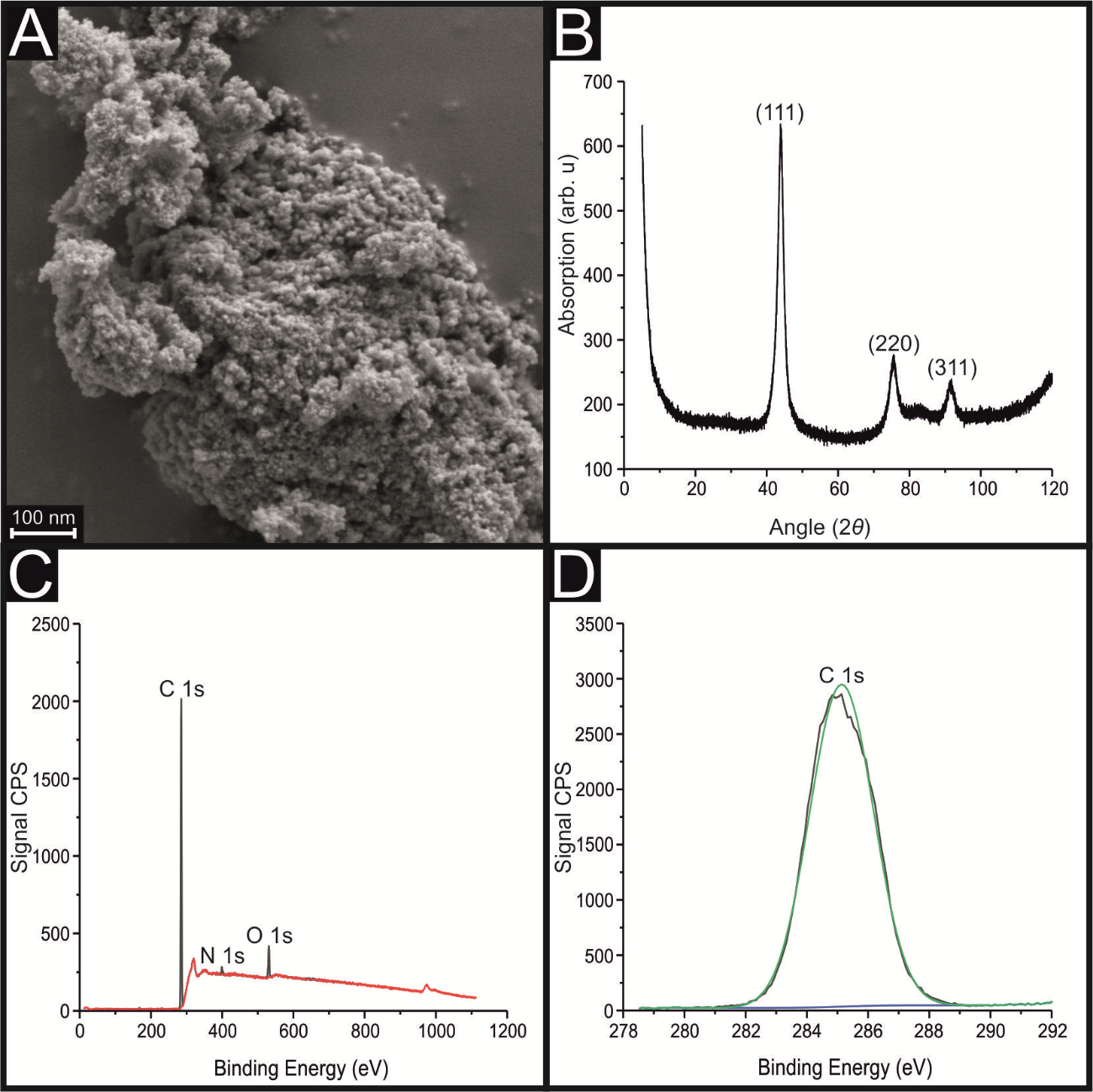


Figure S2. TEM images of the commercially procured NDs. The highlights distinguish the distinct outlines of individual nanodiamonds. Scale bar: 10 nm.


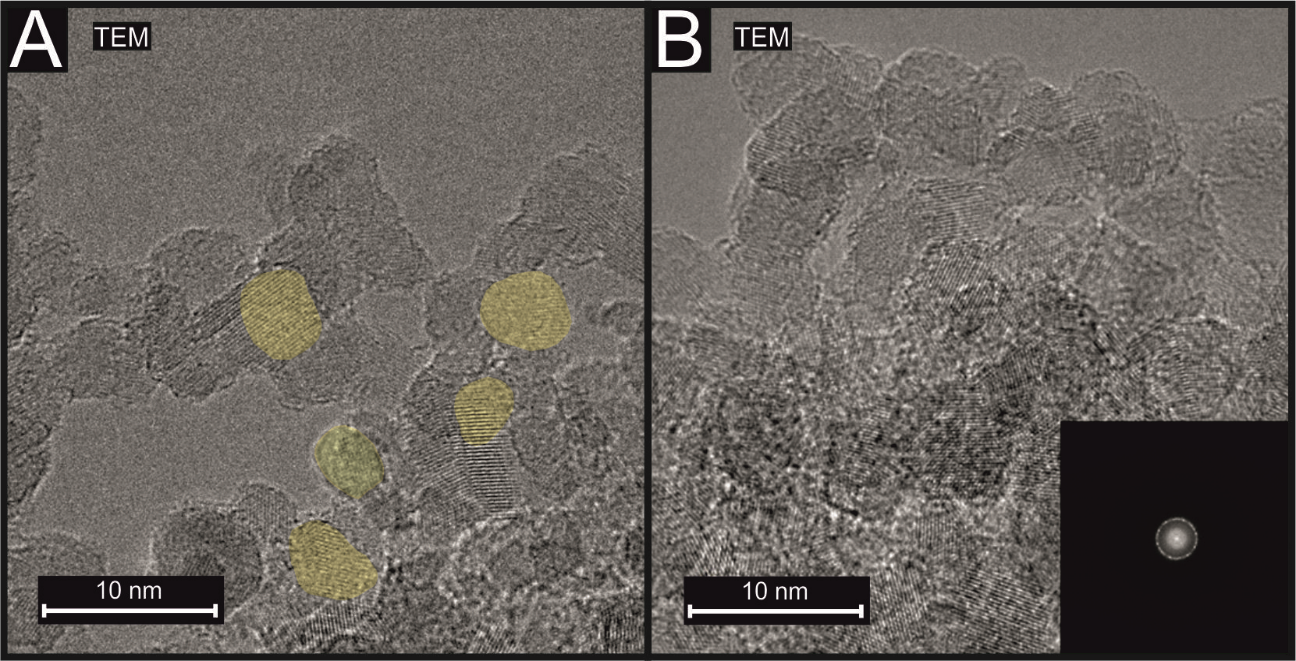


**Figure S3.** Raman spectrum of the commercially sourced nanodiamonds.

**
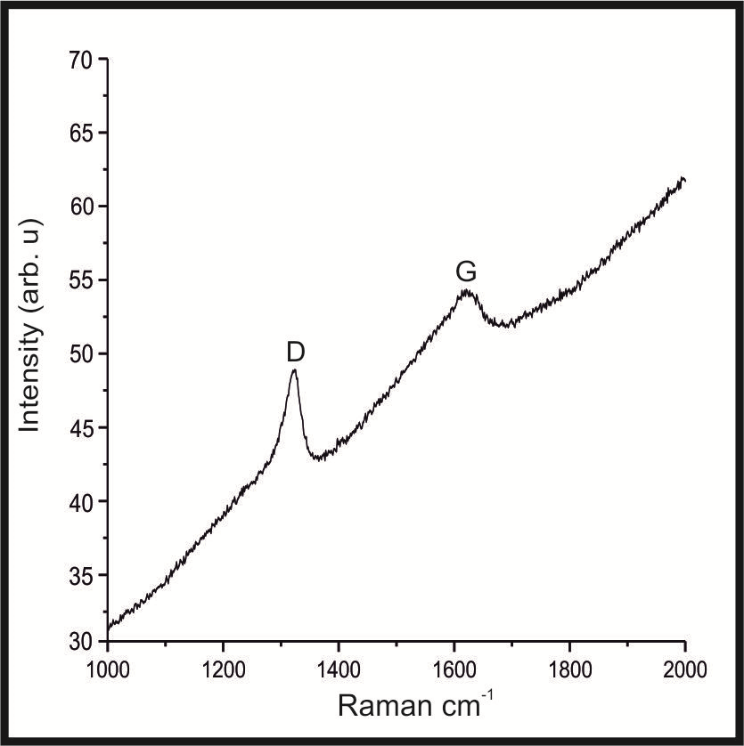
**

**Figure S4.** Typical DPV voltammograms obtained using *ca.* 140 ng cm^−2^ ND/SPEs indicating the effect of pH solution composition evaluated using 50 µM DA and UA. pH values: 4.4 (black), 5.5 (orange), 6.5 (blue), 7.4 (green), and 8.4 (purple). Parameters of DPV: E-pulse = 20 mV; t-pulse = 200 ms; equivalent scan rate = 10 mV s^−1^.


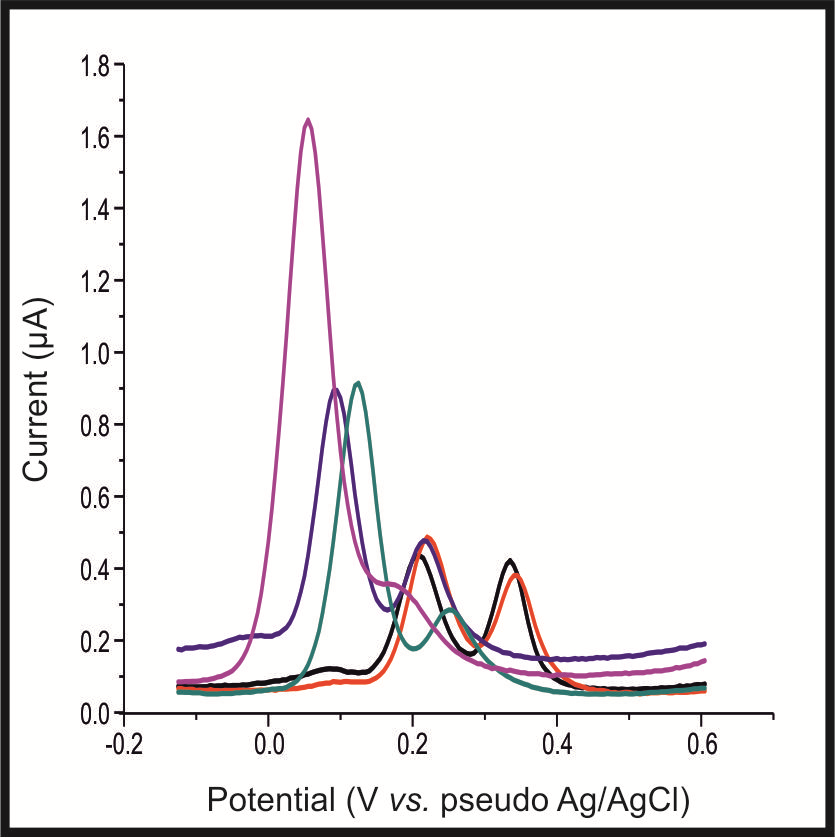


**Figure S5.** A comparison of the electroanalytical outputs for pH 5.5 phosphate buffer (solid line) and pH 5.5 acetate buffer (dotted line). DPVs obtained with a *ca*. 140 ng cm^−2^ ND/SPE in the presence 50 µM DA and 50 µM UA Parameters of DPV; E-pulse: 20 mV; t-pulse: 200 ms; equivalent scan rate: 10 mV s^−1^.

**
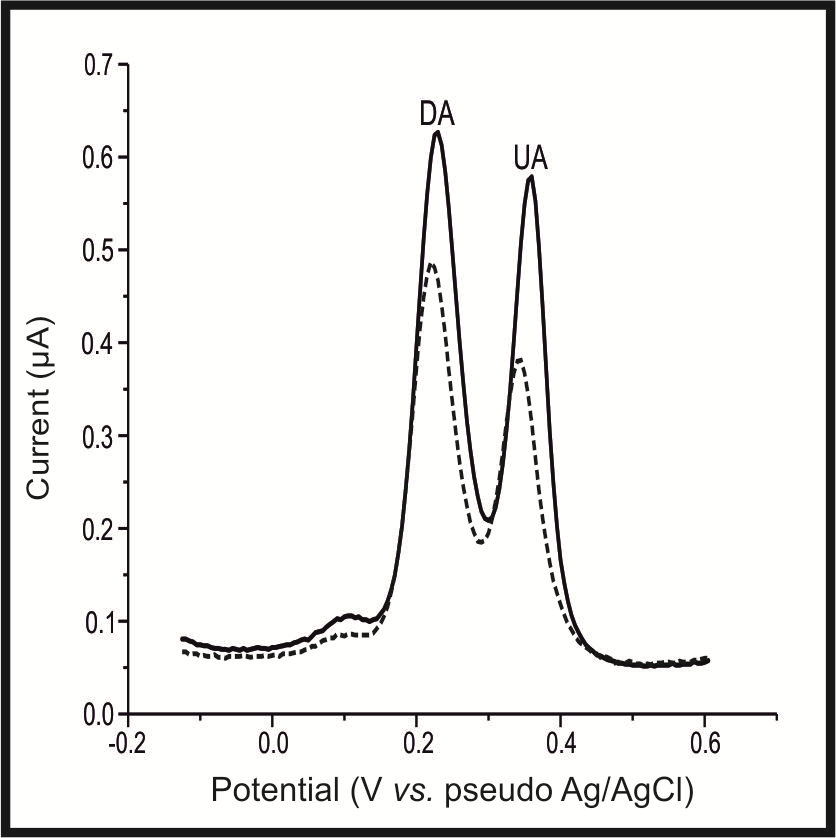
**

**Figure S6.** DPVs obtained using a bare/unmodified SPE (dotted line) and a *ca*. 140 ng cm^−2^ ND/SPE (Solid line) in the presence 50 µM DA and 50 µM UA in 0.10 M acetate buffer (pH 5.5). Parameters of DPV: E-pulse = 20 mV; t-pulse = 200 ms; equivalent scan rate: 10 mV s^−1^.

**
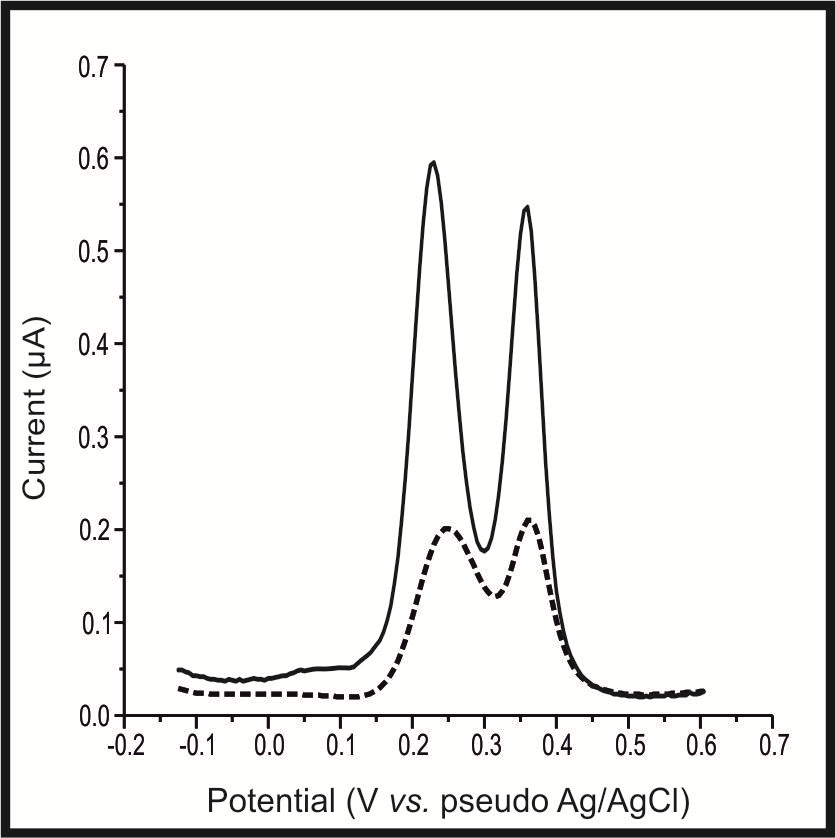
**

**Figure S7.** White light profilometry surface topography maps of bare/unmodified (A) EPPG, (B) GCE and (C) SPE. It is evident that the surface roughness of an SPE is relatively greater than that of the other carbon based electrodes. SQ being the Root mean squared value of the heights over the whole surface, SA being is the arithmetic average values of absolute height values over the whole surface

**
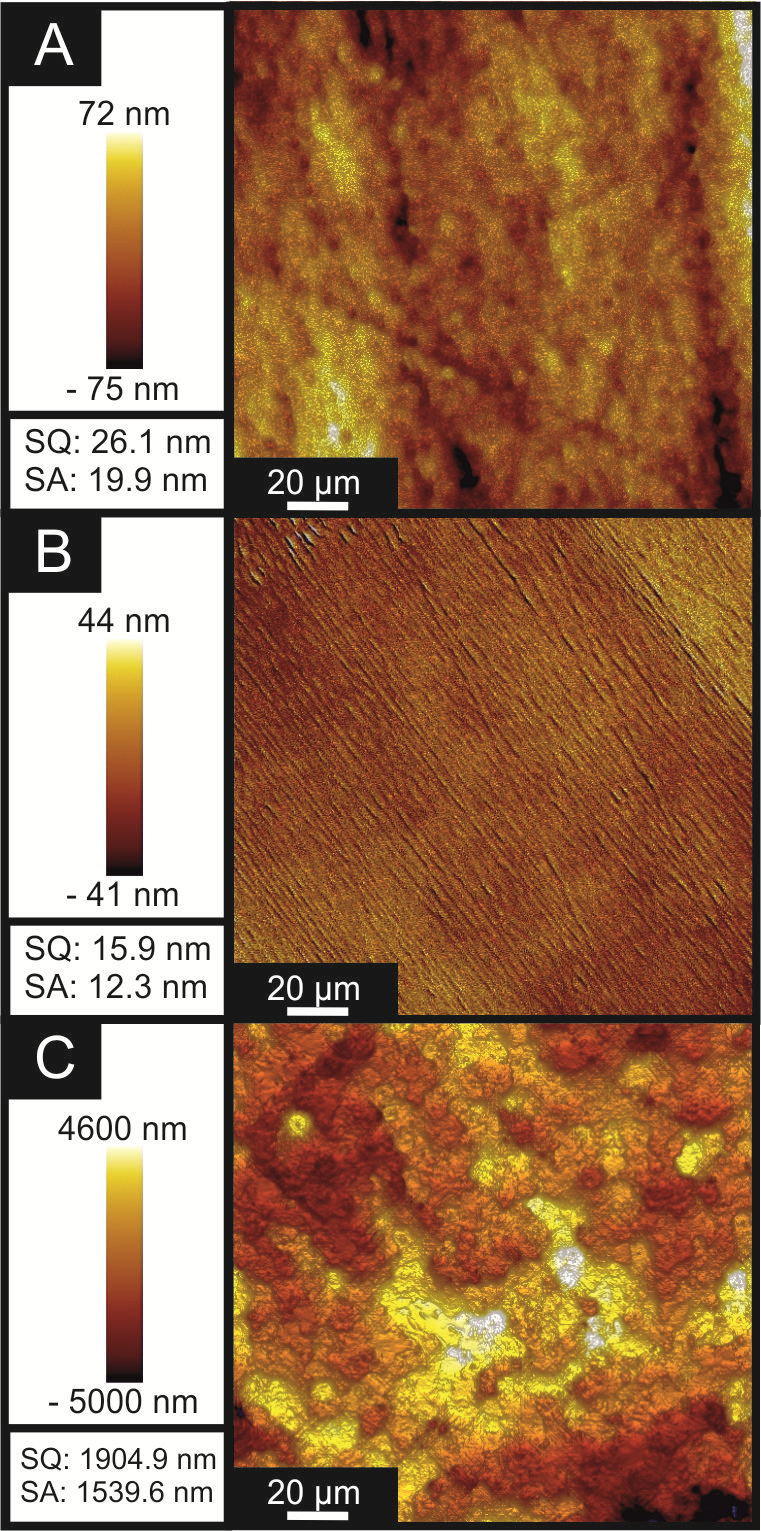
**

**Figure S8.** XPS spectra of the (A) excited and (B) differentiated C KVV Auger peaks for the commercially sourced NDs.

**
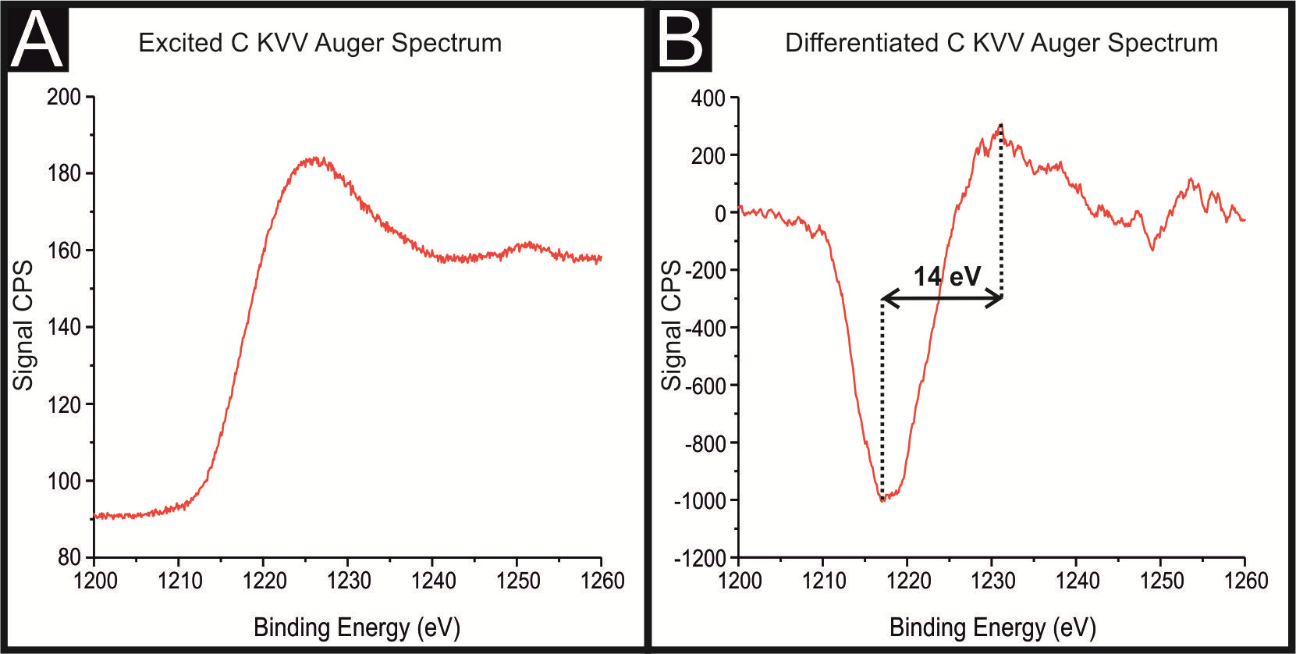
**

**References**

1. Choudry, N. A.; Kampouris, D. K.; Kadara, R. O.; Banks, C. E., Disposable Highly Ordered Pyrolytic Graphite-like Electrodes: Tailoring the Electrochemical Reactivity of Screen Printed Electrodes. *Electrochem. Commun.* **2010,** *12* (1), 6-9.

2. Cumba, L. R.; Smith, J. P.; Brownson, D. A. C.; Iniesta, J.; Metters, J. P.; Carmo., D. R. D.; Banks, C. E., Electroanalytical Detection of Pindolol: Comparison of Unmodified and Reduced Graphene Oxide Modified Screen-Printed Graphite Electrodes. *Analyst* **2015,** *140* (5), 1543-1550.

3. Foster, C. W.; Pillay, J.; Metters, J. P.; Banks, C. E., Cobalt Phthalocyanine Modified Electrodes Utilised in Electroanalysis: Nano-Structured Modified Electrodes vs. Bulk Modified Screen-Printed Electrodes. *Sensors (Basel, Switzerland)* **2014,** *14* (11), 21905-21922.

4. Foster, C. W.; Metters, J. P.; Banks, C. E., Ultra Flexible Paper Based Electrochemical Sensors: Effect of Mechanical Contortion upon Electrochemical Performance. *Electroanalysis* **2013,** *25* (10), 2275-2282.

5. Metters, J. P.; Gomez-Mingot, M.; Iniesta, J.; Kadara, R. O.; Banks, C. E., The Fabrication of Novel Screen Printed Single-Walled Carbon Nanotube Electrodes: Electroanalytical Applications. *Sens. Actuators, B* **2013,** *177*, 1043-1052.

6. Rowley-Neale, S. J.; Brownson, D. A. C.; Smith, G. C.; Sawtell, D. A. G.; Kelly, P. J.; Banks, C. E., 2D Nanosheet Molybdenum Disulphide (MoS2) Modified Electrodes Explored Towards the Hydrogen Evolution Reaction. *Nanoscale* **2015,** *7* (43), 18152-18168.

7. Galdino, F. E.; Foster, C. W.; Bonacin, J. A.; Banks, C. E., Exploring the Electrical Wiring of Screen-Printed Configurations Utilised in Electroanalysis. *Anal. Methods* **2015,** *7* (3), 1208-1214.

8. Rowley-Neale, S. J.; Brownson, D. A. C.; Banks, C. E., Defining the origins of electron transfer at screen-printed graphene-like and graphite electrodes: MoO2 nanowire fabrication on edge plane sites reveals electrochemical insights. *Nanoscale* **2016,** *8* (33), 15241-15251.

9. Innovation in Surface Spectroscopy and Microscopy Systems. <http://www.specs.de/cms/front_content.php?idcat=209> (accessed 20/03/2016).

10. Korepanov, V. I.; Hamaguchi, H.-O.; Osawa, E.; Ermolenkov, V.; Lednev, I. K.; Etzold, B. J. M.; Levinson, O.; Zousman, B.; Epperla, C. P.; Chang, H.-C., Carbon structure in nanodiamonds elucidated from Raman spectroscopy. *Carbon* **2017,** *121*, 322-329.

11. Wang, Z.; Xu, C.; Liu, C., Surface modification and intrinsic green fluorescence emission of a detonation nanodiamond. *J. Mater. Chem. C* **2013,** *1* (40), 6630-6636.

12. Lascovich, J. C.; Scaglione, S., Comparison among XAES, PELS and XPS techniques for evaluation of Sp2 percentage in a-C:H. *Appl. Surf. Sci.* **1994,** *78* (1), 17-23.

13. Lin, L.; Chen, J.; Yao, H.; Chen, Y.; Zheng, Y.; Lin, X., Simultaneous determination of dopamine, ascorbic acid and uric acid at poly (Evans Blue) modified glassy carbon electrode. *Bioelectrochemistry* **2008,** *73* (1), 11-17.

14. Jahani, S.; Beitollahi, H., Selective Detection of Dopamine in the Presence of Uric Acid Using NiO Nanoparticles Decorated on Graphene Nanosheets Modified Screen-printed Electrodes. *Electroanalysis* **2016,** *28* (9), 2022-2028.

15. Rafati, A. A.; Afraz, A.; Hajian, A.; Assari, P., Simultaneous determination of ascorbic acid, dopamine, and uric acid using a carbon paste electrode modified with multiwalled carbon nanotubes, ionic liquid, and palladium nanoparticles. *Microchim. Acta* **2014,** *181* (15), 1999-2008.

16. Qi, S.; Zhao, B.; Tang, H.; Jiang, X., Determination of ascorbic acid, dopamine, and uric acid by a novel electrochemical sensor based on pristine graphene. *Electrochim. Acta* **2015,** *161*, 395-402.

17. Safavi, A.; Maleki, N.; Moradlou, O.; Tajabadi, F., Simultaneous determination of dopamine, ascorbic acid, and uric acid using carbon ionic liquid electrode. *Anal. Biochem.* **2006,** *359* (2), 224-229.
